# Extracellular Matrix and Oxidative Phosphorylation: Important Role in the Regulation of Hypothalamic Function by Gut Microbiota

**DOI:** 10.3389/fgene.2020.00520

**Published:** 2020-06-25

**Authors:** Xunzhong Qi, Xiaogang Zhong, Shaohua Xu, Benhua Zeng, Jianjun Chen, Guangchao Zang, Li Zeng, Shunjie Bai, Chanjuan Zhou, Hong Wei, Peng Xie

**Affiliations:** ^1^Department of Neurology, The First Affiliated Hospital of Chongqing Medical University, Chongqing, China; ^2^Institute of Neuroscience, Chongqing Medical University, Chongqing, China; ^3^Chongqing Key Laboratory of Neurobiology, Chongqing Medical University, Chongqing, China; ^4^Institute of Neuroscience and the Collaborative Innovation Center for Brain Science, Chongqing Medical University, Chongqing, China; ^5^School of Public Health and Management, Chongqing Medical University, Chongqing, China; ^6^Department of Neurology, Yongchuan Hospital of Chongqing Medical University, Chongqing, China; ^7^Department of Laboratory Animal Science, College of Basic Medical Sciences, Army Medical University, Chongqing, China; ^8^Institute of Life Sciences, Chongqing Medical University, Chongqing, China; ^9^Pathogen Biology and Immunology Laboratory, and Laboratory of Tissue and Cell Biology, Experimental Teaching and Management Center, Chongqing Medical University, Chongqing, China; ^10^Department of Nephrology, The Second Affiliated Hospital of Chongqing Medical University, Chongqing, China; ^11^Department of Laboratory Medicine, The First Affiliated Hospital of Chongqing Medical University, Chongqing, China

**Keywords:** major depressive disorder, gut microbiota, hypothalamus, extracellular matrix, oxidative phosphorylation

## Abstract

**Background:**

In previous studies, our team examined the gut microbiota of healthy individuals and depressed patients using fecal microbiota transplantation of germ-free (GF) mice. Our results showed that depression-like and anxiety-like behavioral phenotypes of host mice were increased, but the molecular mechanism by which gut microbiota regulate host behavioral phenotypes is still unclear.

**Methods:**

To investigate the molecular mechanism by which gut microbiota regulate host brain function, adult GF mice were colonized with fecal samples derived from healthy control (HC) individuals or patients with major depressive disorder (MDD). Transcriptomic profiling of hypothalamus samples was performed to detect differentially expressed genes (DEGs). qRT-PCR was used for validation experiments.

**Results:**

Colonization germ-free (CGF) mice had 243 DEGs compared with GF mice. The most enriched KEGG pathways associated with upregulated genes were “protein digestion and absorption,” “extracellular matrix (ECM)-receptor interaction,” and “focal adhesion.” MDD mice had 642 DEGs compared with HC mice. The most enriched KEGG pathways associated with upregulated genes in MDD mice were also “protein digestion and absorption,” “ECM-receptor interaction,” and “focal adhesion.” Meanwhile, the most enriched KEGG pathway associated with downregulated genes in these mice was “oxidative phosphorylation,” and genes related to this pathway were found to be highly correlated in PPI network analysis.

**Conclusion:**

In summary, our findings suggested that regulation of ECM is a key mechanism shared by different gut microbiota and that inhibition of energy metabolism in the hypothalamus by gut microbiota derived from MDD patients is a potential mechanism of behavioral regulation and depression.

## Introduction

The gut microbiota is a complex biological community inhabiting the digestive tract of the host. As an important environmental factor, the gut microbiota plays an important role in regulating the body functions of the host. Previous studies have shown that gut microbiota can regulate intestinal development (Hooper, 2004; Backhed et al., 2005), liver function (Safari and Gerard, 2019), bone development (Schwarzer et al., 2016; Yan et al., 2016), the immune system (Hooper et al., 2012), and host metabolism (Velagapudi et al., 2010; Nicholson et al., 2012). In recent years, studies have also reported that the gut microbiota plays an important regulatory role in brain function, including neurotransmission (Diaz Heijtz et al., 2011), neuroimmunology (Paolicelli et al., 2011; Erny et al., 2015; Mohle et al., 2016), neurotrophic factors (Bercik et al., 2011), blood–brain barrier function (Braniste et al., 2014), axonal development (Luczynski et al., 2016), and host behavioral phenotypes (Diaz Heijtz et al., 2011). Our team also confirmed that, compared with SPF mice, GF mice exhibited anti-anxiety and anti-depression behavioral phenotypes as a result of a lack of gut microbiota stimulation (Zeng et al., 2016; Zheng et al., 2016; Huo et al., 2017). On this basis, the gut microbiota of healthy control (HC) individuals and of patients with MDD were used for fecal microbiota transplantation of GF mice. The results showed increased depression-like and anxiety-like behavioral phenotypes of the host with fecal transplantation from MDD patients and that their metabolic function was also affected (Zheng et al., 2016). This result further confirmed the important role of gut microbiota in brain function and behavioral regulation; however, the molecular mechanism by which gut microbiota regulate host behavioral phenotypes is still unclear.

Activation of the HPA is considered to be a specific reaction against stress. Clinical studies have indicated that hyperfunction of the HPA axis is an important neurobiological characteristic of patients with depressive disorders (Morris et al., 2012). Moreover, the relationship between depression-like behavior and the HPA axis has been widely recognized (Heuser et al., 1996; Nickel et al., 2003; Barden, 2004).

In the presence of various stimuli, metabolism, cardiovascular, and cerebrovascular function and immune responses are regulated by the HPA axis. At the same time, inflammatory responses can also regulate the function of the HPA axis through highly expressed inflammatory factors (Marques-Deak et al., 2005), thus forming a neuro–endocrine–immune regulatory network that participates in the regulation of behavioral phenotypes. The hypothalamus is at the center of this regulatory network.

Previous studies by our team also confirmed that levels of adrenocorticotropic hormone, corticotropin-releasing hormone, cortisol, and aldosterone were significantly increased while nuclear receptor subfamily 3 (group C, member 2, *Nr3c2*) was decreased in the hypothalamus of GF mice stimulated by chronic restraint stress (Huo et al., 2017). This result indicated that gut microbiota can regulate the hypothalamic function of the host. However, the molecular mechanism still needed further analysis. As such, the present study was carried out to investigate the influence of microbiota on the transcriptomic profile of the hypothalamus and to test the hypothesis that microbiota form an integral part of hypothalamic function.

## Experimental Procedures

### Animals

Germ-free mice (Kunming male, aged 4–8 weeks) were provided by the Department of Laboratory Animal Science of the Third Military Medical University (Chongqing, China). All experiments were performed in accordance with the National Institutes of Health Guide for the Care and Use of Laboratory Animals (NIH Publication No. 80-23, revised in 1996) and were approved by the Animal Ethics Committee of Chongqing Medical University.

### Microbiota Colonization

In the colonization protocol, GF mice were placed in cages with bedding and fecal matter from SPF mice and raised next to SPF mice for 3 weeks to allow microbes present in the environment to colonize them (*n* = 15) [colonized germ-free (CGF) mice], as previously demonstrated to be effective for normal microbiota colonization (Clarke et al., 2013).

### Subject Recruitment and Sample Collection

Prior to the collection of human fecal samples, written informed consent forms were obtained from all subjects. The protocols of clinical experimentation were reviewed and approved by the Ethical Committee of Chongqing Medical University (ECCMU). MDD and healthy subjects were recruited, as previously described (Zheng et al., 2012, 2013, 2016). All diagnoses were carried out according to the Structured Psychiatric Interview using DSM-IV-TR criteria. The 17-item version of the observer-rated Hamilton Depression Rating Scale (HDRS) was used to assess depression severity. MDD candidates were excluded on the basis of substance abuse in addition to pregnancy, nursing, or current menstruation for female subjects. HCs were excluded on the basis of a history of systemic medical illness or mental disorders or a family history of any psychiatric disorder. A total of 58 MDD patients and 63 demographically matched HCs were recruited from the psychiatric center and medical examination center of The First Affiliated Hospital at Chongqing Medical University, respectively. The majority of MDD subjects (*n* = 39) were drug-naive, while the remaining MDD subjects (*n* = 19) were being treated with various anti-depressants. All MDD subjects and HCs who were using antibiotics or prebiotics were excluded. The detailed individual demographic and clinical data of the recruited subjects are presented in previous studies (Zheng et al., 2016).

### Fecal Microbiota Transplantation (FMT)

Fecal samples from MDD patients (*n* = 5, male, age 27–61 years) and HCs (*n* = 5, male, age 29–62 years) were used for FMT. The procedures for preparing the fecal samples for microbiota transplantation were as described in previous studies (Koren et al., 2012; Braniste et al., 2014; Zheng et al., 2016). Adult (6–8-week-old) male GF Kunming mice were colonized with pooled samples derived from either MDD patients or HCs. The recipient mice were separately bred in different gnotobiotic isolators to prevent normalization of gut microbiota. Behavioral tests (including OFT, FST, and TST) were performed on weeks 1 and 2 after microbiota transplantation. Cecal samples were collected at the time the mice were killed, and these were immediately snap-frozen in liquid N_2_ and stored at −80°C.

### Behavioral Testing

For the OFT, mice were rested in the testing room for 1 h to acclimate before testing and were then placed at the center of the open field arena for 6 min of exploration. The total distance and distance moved in the internal area were recorded by tracking software and analyzed by SMART 2.5 software. Spontaneous activity of mice was measured during the last 5 min (Deacon, 2006).

For the FST, mice were pre-tested with a 15-min swim 24 h before the formal test and habituated to the testing room 1 h prior to swim testing. During test sessions, mice were placed individually in a Plexiglas cylinder filled with 15 cm of water for 6 min, with the last 5 min scored for immobility. Tracking software and SMART 2.5 software were used to record and analyze all FST experiments (Slattery and Cryan, 2012).

### Quantitative Real-Time PCR (qRT-PCR) Analysis

As previously described, hypothalamus samples were dissected out on ice-cold plate and stored at −80°C before assay. All experimental procedures were in accord with the kit instructions. RNA from the hypothalamus samples was obtained by the TRIzol extraction protocol (Thermo Fisher Scientific, Rockford, IL, United States) and reverse-transcribed with a PrimeScript RT Master Mix Kit (Takara, Otsu, Japan) (*n* = 5). qRT-PCR was carried out with an SYBR Premix Ex Taq II (Takara) on a LightCycler 96 System (Roche, Basel, Switzerland). Nucleotide primers are shown in the Supplementary Material (Supplementary Appendix Table 1). The relative expression levels were calculated by the 2^–ΔΔ*Ct*^ method after the (cycle threshold) Ct value (power amplification knee point) had been obtained. Differences between groups were assessed by Student’s *t*-test at a significance level of *P* < 0.05, with transcript levels normalized relative to β-actin mRNA (housekeeping gene).

### RNA-Seq Analysis

Total RNA was extracted from the tissue using TRIzol^®^ Reagent, and a high-quality RNA sample (OD260/280 = 1.8∼2.2, OD260/230 ≥ 2.0, RIN ≥ 6.5, 28S:18S ≥ 1.0, >10 μg) was used to construct the sequencing library (*n* = 3). The RNA-seq transcriptome library was prepared with the TruSeq^TM^ RNA sample preparation kit from Illumina (San Diego, CA, United States) using 5 μg of total RNA. After being quantified by TBS380, the paired-end RNA-seq sequencing library was sequenced with the Illumina HiSeq 4000. A HiSeq4000 SBS Kit (Illumina, San Diego, CA, United States) was used for transcriptomic profiling experiments according to the standard protocol.

Significance analysis of RNA-seq data with | log2 Fold Change| ≥ 1.5 and *P*-adjust < 0.05 was used to identify DEGs. GO annotations of DEGs were obtained using GO analysis^1^. KEGG pathways with an FDR < 0.05 were selected. Protein–protein interaction (PPI) networks were analyzed using the Search Tool for the Retrieval of Interacting Genes (STRING^2^).

## Results

### Microbiota of MDD Patients Increased Depression-Like Behaviors in GF Recipient Mice

The results of behavioral tests were reported in our previous articles (Zheng et al., 2016; Qi et al., 2019). Briefly, compared with GF mice, CGF mice showed no significant change in behavioral test performance (Qi et al., 2019). Compared with HC mice, MDD mice displayed less exploration of the center of the open field in the OFT test and had more time of immobility in the FST (Zheng et al., 2016). These results indicated that the microbiota of MDD patients can increase depression-like behavior in GF recipient mice.

### Gut Microbiota Promote Expression of Extracellular Matrix (ECM)-Related Genes in the Hypothalamus

**FIGURE 1:**
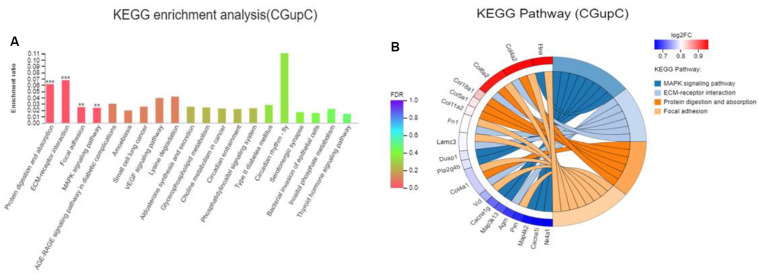
Gut microbiota promote the expression of extracellular matrix-related genes in the hypothalamus. **(A)** Histogram representing KEGG enrichment analysis of expressed genes upregulated in colonized germ-free (CGF) versus uncolonized germ-free (GF) mice. ***FDR < 0.001, **FDR < 0.01; **(B)** Chord diagram representing KEGG enrichment analysis of up-regulated genes in CGF versus GF mice. CGupC, Genes upregulated in CGF mice compared with GF mice; CGupG, Genes downregulated in CGF mice compared with GF mice.

**FIGURE 2:**
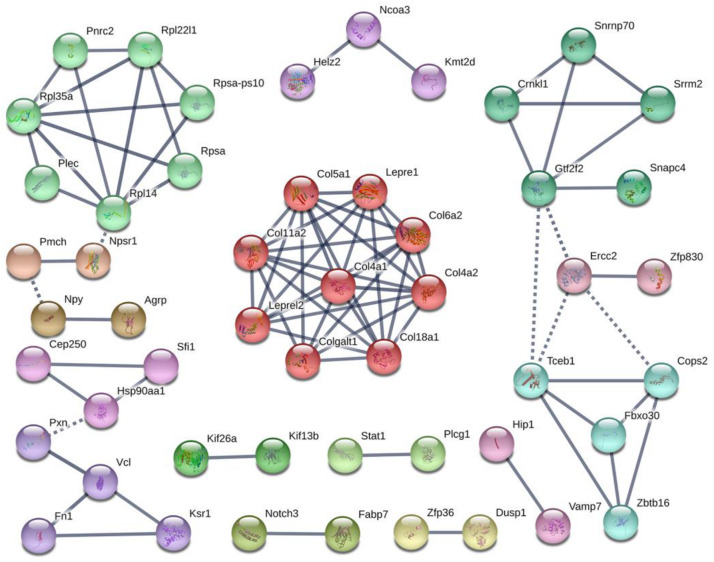
Protein–protein interaction network construction and module analysis of genes differentially expressed between colonized germ-free (CGF) and uncolonized germ-free (GF) mice.

Colonization germ-free mice showed 243 DEGs compared with GF mice, including 172 upregulated (CGupC) and 71 downregulated (CGupG) genes. The most enriched KEGG pathways associated with up-regulated genes (FDR < 0.05) were “protein digestion and absorption” (map04974, FDR = 0.000), “ECM-receptor interaction” (map04512, FDR = 0.000), “focal adhesion” (map04510, FDR = 0.006), and “MAPK signaling” (map04010, FDR = 0.006) (Figure 1) (Supplementary Appendix Table 2). ECM-receptor interaction and focal adhesion are both associated with ECM. Indeed, all six genes (*Col4a1*, *Col4a2*, *Col5a1*, *Col6a2*, *Col11a2*, and *Col18a1*) enriched in the protein digestion and absorption pathway were ECM subtypes. Potential relationships among all DEGs were predicted based on an interaction score >0.9 in the STRING database. The most important PPI network modules are shown in the center of Figure 2. Nine hub genes (*Col4a1*, *Col4a2*, *Col5a1*, *Col6a2*, *Col11a2*, *Col18a2*, *Colgalt1*, *lepre1*, and *lepre2*) with the highest degree of connectivity were all associated with ECM. These results suggested that gut microbiota can promote the expression of ECM-related genes in the hypothalamus of the host.

### Gut Microbiota Derived From MDD Patients Promoted Expression of ECM-Related Genes in the Hypothalamus

**FIGURE 3:**
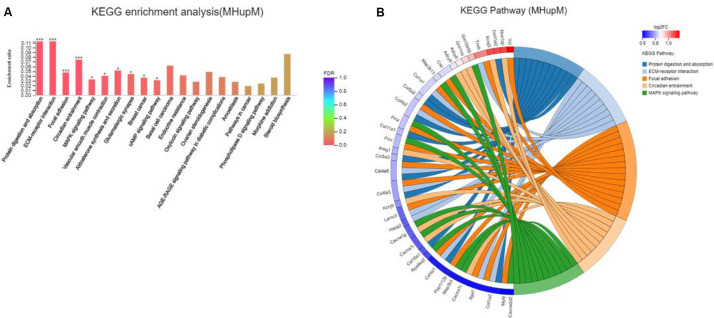
Gut microbiota derived from patients with major depressive disorder (MDD) promoted the expression of extracellular matrix-related genes in the hypothalamus. **(A)** Histogram representing KEGG enrichment analysis of expressed genes upregulated in MDD versus HC. ***FDR < 0.001, *FDR < 0.05; **(B)** Chord diagram representing KEGG enrichment analysis of genes upregulated in MDD versus HC. MHupM: Genes upregulated in MDD mice compared with HC mice.

Major depressive disorder mice had 642 DEGs compared with HC mice, including 336 up-regulated (MHupM) and 306 downregulated (MHupH) genes. The most enriched KEGG pathways associated with up-regulated genes (MHupM, FDR < 0.001) were “protein digestion and absorption” (map04974, FDR = 0.000), “ECM-receptor interaction” (map04512, FDR = 0.000), “focal adhesion” (map04510, FDR = 0.000), and “circadian entrainment” (map04713, FDR = 0.000, Figure 3) (Supplementary Appendix Table 3). The first three enriched KEGG pathways in MHupM were completely consistent with those identified in CGupC. Indeed, ECM-receptor interaction and focal adhesion pathways were both associated with ECM, and all 11 genes (*Col18a1*, *Col4a5*, *Col6a3*, *Col5a2*, *Col4a1*, *Col1a2*, *Col4a2*, *Col11a2*, *Col11a1*, *Col1a1*, and *Col5a3*) enriched in the protein digestion and absorption pathway were related to ECM.

**FIGURE 4:**
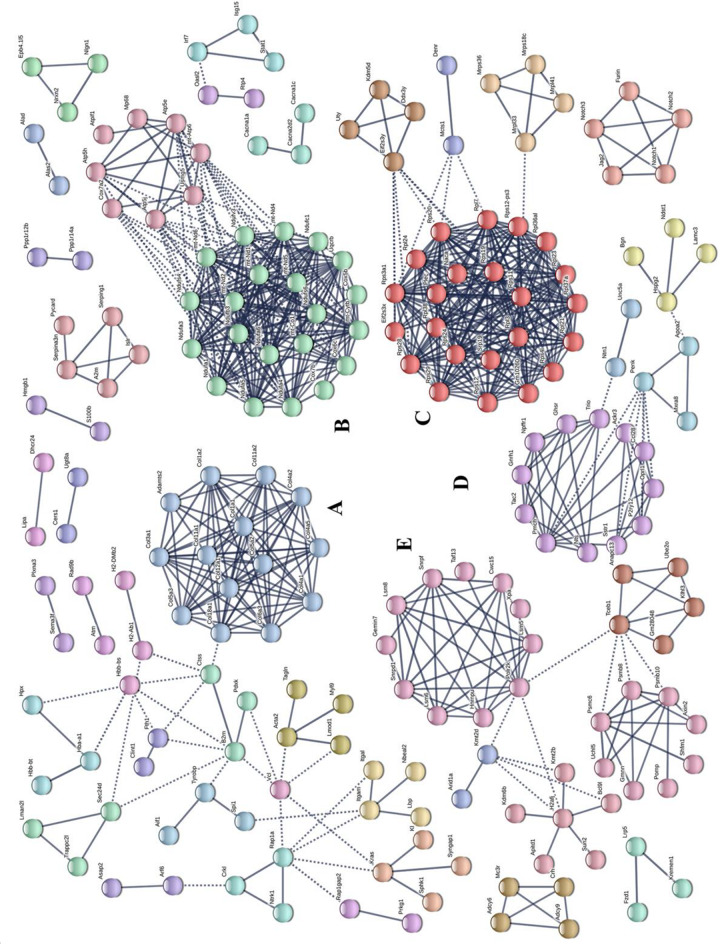
Protein–protein interaction network construction and module analysis of genes differentially expressed between patients with major depressive disorder (MDD) and healthy controls (HC). **(A)** Extracellular matrix; **(B)** Oxidative phosphorylation; **(C)** Ribosome; **(D)** Neuroactive ligand-receptor interaction; **(E)** Spliceosome.

Potential relationships among all DEGs were predicted based on an interaction score >0.9 in the STRING database. The most important PPI network modules are shown in the center of Figure 4. Fourteen hub genes (*Col1a1*, *Col1a2*, *Col3a1*, *Col4a1*, *Col4a2*, *Col4a5*, *Col5a2*, *Col5a3*, *Col6a3*, *Col11a1*, *Col11a2*, *Col12a1*, *Col18a1*, and *Adamts2*) with a high degree of connectivity in sub-network A were all associated with ECM. These results also suggested that gut microbiota derived from MDD patients can promote the expression of ECM-related genes in the hypothalamus of the host.

### ECM Plays a Critical Role in the Regulation of Hypothalamic Function by Gut Microbiota

**FIGURE 5:**
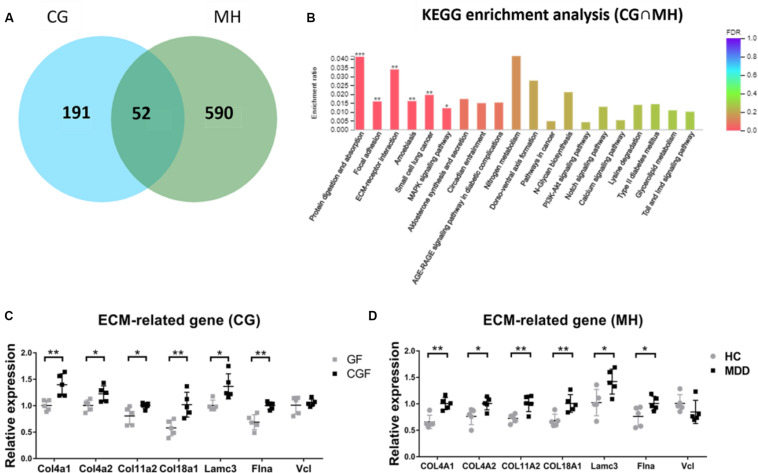
**(A)** Venn diagram of 52 overlapping differentially expressed genes (DEGs) from CG and MH; **(B)** Histogram representing KEGG enrichment analysis of overlapping DEGs. ***FDR < 0.001, **FDR < 0.01, *FDR < 0.05. **(C)** Gut microbiota promoted the expression of extracellular matrix (ECM)-related genes in the hypothalamus (*n* = 5); **(D)** Gut microbiota derived from patients with major depressive disorder promoted the expression of ECM-related genes in the hypothalamus (*n* = 5). **P < 0.01, *P < 0.05 (assessed by Student’s *t*-test); CG, DEGs between CGF mice and GF mice; MH, DEGs between MDD mice and HC mice; CG∩MH, Overlapping DEGs from CG and MH.

As there were so many similarities between MHupM and CGupC in KEGG enrichment, the 52 overlapping DEGs in MH and CG were analyzed by KEGG enrichment (Figure 5A). The results showed that the most enriched KEGG pathways were also “protein digestion and absorption” (map04974, FDR = 0.000) (Col4a1, Col4a2, Col11a2, Col18a1), “ECM-receptor interaction” (map04512, FDR = 0.001) (Col4a1, Col4a2, Lamc3), and “focal adhesion” (map04510, FDR = 0.000) (Col4a1, Col4a2, Vcl, Lamc3, Flna), which were completely consistent with those identified in MHupM and CGupC (Supplementary Appendix Table 4 and Figure 5B). Seven genes involved in these three pathways were selected for validation by qRT-PCR, and the expression of six genes was consistent with the sequencing results [Figure 5C, *Col4a1* (*F*1,8 = 3.166, *P* = 0.005), *Col4a2* (*F*1,8 = 0.609, *P* = 0.027), *Col11a2* (*F*1,8 = 12.551, *P* = 0.042), *Col18a1* (*F*1,8 = 1.042, *P* = 0.008), *Lamc3* (*F*1,8 = 3.557, *P* = 0.014), *Flna* (*F*1,8 = 1.377, *P* = 0.002), *Vcl* (*F*1,8 = 12.790, *P* = 0.587)], [Figure 5D, *Col4a1* (*F*1,8 = 0.000, *P* = 0.002), *Col4a2* (*F*1,8 = 0.234, *P* = 0.026), *Col11a2* (*F*1,8 = 0.645, *P* = 0.007), *Col18a1* (*F*1,8 = 0.492, *P* = 0.008), *Lamc3* (*F*1,8 = 0.018, *P* = 0.031), *Flna* (*F*1,8 = 1.674, *P* = 0.042), *Vcl* (*F*1,8 = 0.292, *P* = 0.223)]. These results confirmed the credibility of the RNA-seq results and also verified that the ECM plays a critical role in the regulation of hypothalamic function by gut microbiota.

### Gut Microbiota Derived From MDD Patients Reduced Expression of Oxidative Phosphorylation-Related Genes in the Hypothalamus

**FIGURE 6:**
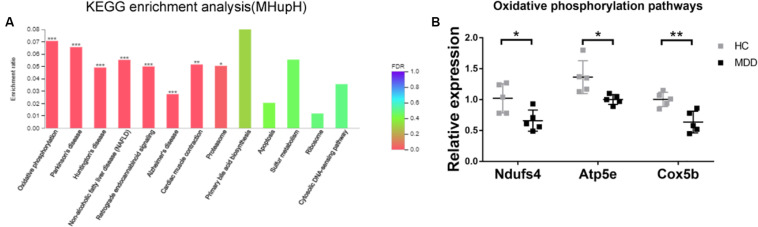
**(A)** Histogram representing KEGG enrichment analysis of genes downregulated in mice colonized with gut microbiota from patients with major depressive disorder (MDD) versus from healthy controls (HC). ***FDR < 0.001, **FDR < 0.01, *FDR < 0.05; **(B)** Gut microbiota derived from MDD patients reduce the expression of genes related to oxidative phosphorylation in the hypothalamus (*n* = 5). **P < 0.01, *P < 0.05 (assessed by Student’s *t*-test).

In KEGG enrichment analysis, the most enriched pathway associated with downregulated genes (MHupH) was “oxidative phosphorylation” (map00190, FDR < 0.000, Figure 6A) (Supplementary Appendix Table 5), and genes related to this pathway were highly correlated in PPI networks (Figure 4B). Genes representing three different parts of the oxidative phosphorylation pathway were selected for qRT-PCR verification. The results indicated that the expression of three genes [NADH dehydrogenase complex: *Ndufs4* (*F*1,8 = 1.139, *P* = 0.025), ATP synthase: *Atp5e* (*F*1,8 = 2.062, *P* = 0.019), and cytochrome C oxidase: *Cox5b* (*F*1,8 = 2.284, *P* = 0.004), Figure 6B] was downregulated in MDD mice, consistent with the RNA-seq results. These results revealed that gut microbiota derived from MDD patients can reduce the expression of genes related to oxidative phosphorylation of the hypothalamus.

For fecal microbiota transplantation in this study, GF mice were colonized with fecal samples via the fecal matter gavage method (MDD mice and HC mice), which is a common method for fecal transplantation (Braniste et al., 2014). We did not perform this operation on the mice of the GF group that was used as the control group for CGF mice. Considering that gavage is a damaging stimulus and may affect the behavioral test results (Gacias et al., 2016), we have not performed a comparison between HC and GF in this experiment.

## Discussion

The ECM is a three-dimensional network of extracellular macromolecules that provides structural and functional support for cells (Theocharis et al., 2016). ECM components are synthesized by resident cells and secreted to the outside of the cell. by exocytosis, where they form a chain network with extracellular fibrin and mucopolysaccharide. Primary ECM components include proteoglycans, proteins, and extracellular vesicles. Interactions between cells and the ECM are mediated, directly or indirectly, by integrins, proteoglycans, CD36, and other surface molecules. The formation of a specific structure at the point of contact between the cell and ECM is called a focal adhesion (Chen et al., 2003; Zaidel-Bar et al., 2004). In this structure, actin is anchored to transmembrane receptors of the integrin family by a multi-molecular complex, thereby forming a structural connection between membrane receptors and the actin cytoskeleton. Local adhesion of relevant components changes cell shape and gene expression by regulating the actin cytoskeleton. Many cytokines and receptors are involved in this process (Riveline et al., 2001; Zamir and Geiger, 2001). In the intercellular environment, ECM is involved in intercellular information transmission, cell adhesion, and regulation of cellular physiological functions such as cell differentiation; moreover, it plays an important regulatory role in cell and tissue functions (Abedin and King, 2010; Michel et al., 2010; Bonnans et al., 2014).

Compared with the control group in this study, gut microbiota derived from both normal mice and MDD patients could promote the expression of genes related to protein digestion and absorption, ECM-receptor interaction, and focal adhesion pathways. Meanwhile, enriched genes in the protein digestion and absorption pathway were all related to ECM components. These results suggested that gut microbiota activated function of the hypothalamic ECM and may regulate hypothalamic function by regulating the ECM. Therefore, ECM plays a critical role in the regulation of hypothalamic function by gut microbiota.

Seven genes involved in protein digestion and absorption, ECM-receptor interaction, and focal adhesion pathways were selected for validation. The expression of six genes was consistent with the sequencing results, thus confirming the reliability of our sequencing results. In addition, the results demonstrated that intervention with gut microbiota could promote expression of *Col4a1*, *Col4a2*, *Col18a1*, *Col11a2*, *Lamc3*, and *Flna* in the host hypothalamus. *Col4a1* and *Col4a2*, both members of the type IV collagen family and major components of the basement membrane, are involved in the construction of ECM and participate in the regulation of various cellular physiological processes, such as cell differentiation and angiogenesis, by binding of integrin α1β1 through their common NC1 domain (Kurkinen et al., 1987; Peissel et al., 1995; Sertie et al., 2000; Kuo et al., 2001; Than et al., 2002; Turner et al., 2015). *Col18a1* is an ECM protein that regulates ECM-dependent motion and morphological changes of cells through its non-collagen domain (Sertie et al., 2000; Kuo et al., 2001). *Col11a2*, a small collagen fiber involved in the construction of ECM, plays an important role in the process of fiber formation by controlling the transverse growth of type II collagen fibrils (Gaudet et al., 2011; Naba et al., 2017). These four collagens are the main components of ECM and directly or indirectly affect a variety of cellular physiological functions. Lamc3, an ECM glycoprotein that binds to cells through high-affinity receptors, interacts with other components of the ECM to mediate important physiological activities such as cell adhesion, morphological changes, and migration; it also participates in astrocyte development (Pinzon-Duarte et al., 2010). Flna is an actin-binding protein involved in the anchoring of actin cytoskeletal membrane proteins. Recombination of the actin cytoskeleton is regulated by interactions of Flna with integrins, transmembrane receptor complexes, and secondary messengers, making it an important protein for adjustments in cell morphology (Feng et al., 2006; Falet et al., 2010). Our results confirmed that gut microbiota derived from HC mice and depressed patients could both promote the expression of collagens Col4a1, Col4a2, Col18a1, and Col11a2, the glycoprotein Lamc3, and the actin-binding protein Flna in ECM, indicating that gut microbiota could affect multiple ECM components, thus regulating cell morphology and function.

Synthesis of ECM is mainly regulated by cytokines, and inflammatory factors play an important role in this process. Studies have shown that interleukin 1 (IL-1) is critical to ECM remodeling during acute inflammatory responses in brain tissue, and both IL-6 and tumor necrosis factor (TNF) are involved in the regulation of ECM (Summers et al., 2010, 2013; Tarassishin et al., 2014). Studies have also shown that interference of gut microbiota can regulate the expression of inflammatory factors in the brain, such as IL-6, IL-1β, and TNF-α (Jang et al., 2018; Hosseinifard et al., 2019), indicating that gut microbiota can regulate ECM components and functions, thus affecting the function of cells by regulating neuroinflammation in the hypothalamus.

In the present study, hypothalamic tissue of MDD mice displayed lower expression of genes related to oxidative phosphorylation pathways compared with HC mice. Oxidative phosphorylation, a process of energy metabolism by which ATP is produced in the mitochondria of eukaryotes, provides a direct source of energy for the body that is essential for its function. In this current study, 26 genes related to oxidative phosphorylation were inhibited by gut microbiota derived from MDD patients. Three genes, *Ndufs4*, *Atp5e*, and *Cox5b*, were selected for verification by qRT-PCR, which confirmed the sequencing results. *Ndufs4* (NADH dehydrogenase [ubiquinone] iron-sulfur protein 4, mitochondrial NDUFS4) is an NADH-ubiquinone oxidoreductase subtype of complex I (van den Heuvel et al., 1998), which is the first multi-subunit enzyme complex in the mitochondrial respiratory chain and plays an important role in cell ATP production. Indeed, *Ndufs4* mutation directly affects the function of NADH-ubiquinone oxidoreductase (Haack et al., 2012; Zong et al., 2013). In addition to *Ndufs4*, DEGs with similar functions, including *Ndufa1*, *Ndufa5*, *Ndufa6*, *Ndufb3*, *Ndufs4*, *Ndufs6*, and *Ndufv3*, were also inhibited by gut microbiota derived from MDD patients. The result was an inhibitory effect on NADH-ubiquinone oxidoreductase in the hypothalamus of host mice.

As an ATP synthase subtype, Atp5e is involved in the ATP synthesis process catalyzed by ADP (Tu et al., 2000) and is an important regulatory enzyme for ATP synthesis. Genetic mutation of Atp53 reportedly causes mitochondrial diseases (Mayr et al., 2010; Hurtado-Lopez et al., 2015). Cox5b, a cytochrome C oxidase subtype, is responsible for electron transfer of cytochrome C and ATP synthesis driven by an electrochemical gradient. It is the terminal enzyme of the mitochondrial respiratory chain (Beauchemin et al., 2001; Zong et al., 2013) and plays an important role in ATP synthesis. But even more importantly, Cox5b is a candidate hippocampal biomarker of susceptibility and resilience to stress in a rat model of depression (Henningsen et al., 2012). In addition to Atp5e and Cox5b, DEGs with similar functions include Atp5h, Atp5j, Atp6v1g2, Cox6c, and Cox7a2, which were also inhibited by gut microbiota derived from MDD patients. The results indicated a modulatory effect on ATP synthesis from ADP and cytochrome C function in the host hypothalamus. In a previous study, our team showed that MDD mice exhibited a depression-like behavioral phenotype compared with HC mice, suggesting that the gut microbiota of depressed patients may be a contributing factor to the development of depressive disorders. In this study, expression of genes in the oxidative phosphorylation pathway was inhibited by the gut microbiota of depressed patients, consistent with the depression-like behavioral phenotypes of MDD mice. These results indicated that the inhibition of hypothalamic oxidative phosphorylation pathways may be a potential mechanism by which the gut microbiota of depressed patients regulates host behavioral phenotypes.

In our previous studies, there were significant differences in gut microbiotic composition between MDD patients and HCs. Specifically, MDD patients were characterized by significant changes in the relative abundance of firmicutes, actinomycetes, and bacteroidetes (Zheng et al., 2016). It has been reported that the gut microbiotic composition was altered by Duodenum-jejunum gastric bypass (DJB), with an increased proportion of firmicutes and decreased actinobacteria in the ileum after surgery. The increased BDNF protein levels in hypothalamus may result from this microbiota change after DJB surgery (Jiang et al., 2016). High-fat diet (HFD) diminished the relative abundance of Bacteroidetes and increased the relative abundance of Firmicutes and Cyanobacteria. It also decreased the expression of NPY in hypothalamus (Hassan and Mancano, 2019). These studies suggested that changes in the relative abundance of firmicutes, actinomycetes, and bacteroidetes may cause changes in hypothalamus function, which may be associated with the expression of ECM-related genes and oxidative phosphorylation-related genes in the hypothalamus. However, the majority of carbohydrate metabolic pathways were disrupted in MDD mice, suggesting a disturbance in energy metabolism that may account for the results of previous studies showing a dramatic protein change in energy mobilization in hypothalamus of chronic unpredictable mild stress (CUMS) mice (Rao et al., 2016). In this study, the process of hypothalamic oxidative phosphorylation was inhibited by gut microbiota of depressed patients, suggesting a reduction in energy synthesis in the hypothalamus that was consistent with previous experiments and reports (Rao et al., 2016). Therefore, this experiment further confirmed that the inhibition of energy metabolism in the hypothalamus by gut microbiota is a potential mechanism of behavioral regulation and depression.

## Conclusion

In summary, our findings suggested that, compared with the control group, gut microbiota derived from either mice or MDD patients could promote the expression of genes associated with ECM, suggesting that regulation of ECM is a key process shared by different gut microbiota. Moreover, our experiments further confirmed that inhibition of energy metabolism in the hypothalamus by gut microbiota derived from MDD patients is a potential mechanism of behavioral regulation and depression.

## Data Availability Statement

The RNA-seq data used to support the findings of this study have been deposited in the SRA repository under accession number PRJNA637458 (https://www.ncbi.nlm.nih.gov/sra/PRJNA637458).

## Ethics Statement

The studies involving human participants were reviewed and approved by the Ethical Committee of Chongqing Medical University. The patients/participants provided their written informed consent to participate in this study. The animal study was reviewed and approved by the Animal Ethics Committee of Chongqing Medical University. Written informed consent was obtained from the individual(s) for the publication of any potentially identifiable images or data included in this article.

## Author Contributions

XQ, HW, and PX: study concept and design. XQ, XZ, SX, and BZ: performing the experiments. GZ, JC, LZ, SB, and CZ: experimental technical guidance. XQ, XZ, and SX: data analysis and manuscript drafting. All authors reviewed and approved the manuscript prior to its submission.

## Conflict of Interest

The authors declare that the research was conducted in the absence of any commercial or financial relationships that could be construed as a potential conflict of interest.

## Footnotes

1 www.geneontology.org

2 http://string-db.org/

## Abbreviations

CGF: Colonization germ-free
DEGs: Differentially expressed genes
ECM: Extracellular Matrix
FDR: False Discovery Rate
FST: Forced swimming test
GF: Germ-free
GO: Gene Ontology
HPA: Hypothalamic–pituitary–adrenal axis
KEGG: Kyoto encyclopedia of genes and genomes
MDD: Major depressive disorder
OFT: Open field test
PPI: Protein–protein interaction network
SPF: Specific pathogen-free.
