# Supplementary material for: Extracellular Matrix and Oxidative Phosphorylation: Important Role in the Regulation of Hypothalamic Function by Gut Microbiota
Source: Front Genet. 2020 Jun 25;11:520. doi: 10.3389/fgene.2020.00520 (PMC7330020; doi:10.3389/fgene.2020.00520)
Supplement: Supplementary file 1 [file Data_Sheet_1.PDF]

Appendix Table 1. Nucleotide primers used for quantitative real-time polymerase chain reaction

Accession numbers	Oligo name	Oligonucleotide sequence (5 ´to 3 ΄)
NM_009931.2	Mouse Col4a1 F	CTGGCACAAAAGGGACGAG
	Mouse Col4a1 R	ACGTGGCCGAGAATTTCACC
NM_009932.4	Mouse Col4a2 F	GACCGAGTGCGGTTCAAAG
	Mouse Col4a2 R	CGCAGGGCACATCCAACTT
NM_001317722.1	Mouse Col11a2 F	GAAGCAGCCCACTGAGTCTC
	Mouse Col11a2 R	CCTCCCCATATTCCTCTGCCT
NM_001109991.1	Mouse Col18a1 F	GTGCCCATCGTCAACCTGAA
	Mouse Col18a1 R	GACATCTCTGCCGTCAAAAGAA
NM_011836.4	Mouse Lamc3 F	TGCCTGGATTCCACTCACTCA
	Mouse Lamc3 R	CAGGGCGGTATCTGTCACA
NM_001290421.1	Mouse Flna F	TCCCCAACCGGGCAAATATG
	Mouse Flna R	CTGGCTACCCTGAGGATAGTT
NM_010887.2	Mouse Ndufs4 F	CTGCCGTTTCCGTCTGTAGAG
	Mouse Ndufs4 R	TGTTATTGCGAGCAGGAACAAA
NM_025983.3	Mouse Atp5e F	CAGGCTGGACTCAGCTACATC
	Mouse Atp5e R	GTTCGCTTTGAACTCGGTCTT
NM_009942.2	Mouse Cox5b F	TTCAAGGTTACTTCGCGGAGT
	Mouse Cox5b R	CGGGACTAGATTAGGGTCTTCC
NM_009502	Mouse Vcl -F	GAGGCTGAACTGCTTCAATCA
	Mouse Vcl -R	CCAGATTTGACGAGGTGCCTA
NM_007393.5	Mouse β-actin-F	AGAGGGAAATCGTGCGTGAC
	Mouse β-actin-R	CAATAGTGATGACCTGGCCGT

Appendix Table 2：KEGG enrichment analysis of up-regulated expressed genes between CGF and GF mice（FDR<0.05）.

Pathway id	Description	Molecules
**map04974**	Protein digestion and absorption	Col18a1,Col4a1,Col4a2,Col11a2,Col5a1, Col6a2
**map04512**	ECM-receptor interaction	Col4a1,Col4a2,Fn1,Lamc3,Agrn,Col6a2
**map04510**	Focal adhesion	Pxn,Col4a1,Col4a2,Fn1,Vcl,Lamc3,Col6a2 Flna
**map04010**	MAPK signaling pathway	Map3k13,Cacna1g,Pla2g4b,Dusp1,Nr4a1 Cacna1i,Map4k2,Flna

Appendix Table 3：KEGG enrichment analysis of up-regulated expressed genes between MDD and HC mice (FDR<0.05).

Pathway id	Description	Molecules
map04974	Protein digestion and absorption	Col18a1,Col4a5,Col6a3,Col5a2,Col4a1,Col1a2 Col4a2,Col11a2,Col11a1,Col1a1,Col5a3
map04512	ECM-receptor interaction	Col4a5,Col6a3,Hspg2,Col4a1,Col1a2,Col4a2 Tnxb,Itga7,Col1a1
map04510	Focal adhesion	Col4a5,Col6a3,Crkl,Col4a1,Ppp1r12b,Col1a2 Col4a2,Vcl,Flnc,Lamc3,Tnxb,Myl9,Itga7,Flna Col1a1
map04713	Circadian entrainment	Adcy9,Nos1ap,Cacna1g,Prkg1,Cacna1c,Kcnj9 Cacna1i,Kcnj3,Adcy6,Grin1os
map04010	MAPK signaling pathway	Map3k13,Cacna1g,Crkl,Cacna1c,Gm15459 Map3k3,Rps6ka2,Cacna2d2,Cacna1i,Flnc,Flna
map04270	Vascular smooth muscle contraction	Adcy9,Prkg1,Cacna1c,Ppp1r12b,Myl9,Acta2 Adcy6,Arhgef11
map04925	Aldosterone synthesis and secretion	Adcy9,Cacna1g,Cacna1c,Scarb1,Cacna1i,Adcy6
map04724	Glutamatergic synapse	Adcy9,Grik3,Cacna1c,Kcnj3,Adcy6,Grin1os
map05224	Breast cancer	Notch3,Axin2,Fzd1,Notch2,Wnt6,Jag2,Lrp5
map04024	cAMP signaling pathway	Ghsr,Adcy9,Sstr1,Cacna1c,Hcn4,Myl9,Adcy6 Grin1os

Appendix Table 4：KEGG enrichment analysis of overlapping DEGs from CG and MH mice（FDR<0.05）.

Pathway id	Description	Molecules
map04974	Protein digestion and absorption	**Col4a1, Col4a2, Col11a2, Col18a1**
map04510	Focal adhesion	**Col4a1, Col4a2, Vcl, Lamc3, Flna**
map04512	ECM-receptor interaction	**Col4a1, Col4a2, Lamc3**
map05146	Amoebiasis	Col4a1, Col4a2, Vcl, Lamc3
map05222	Small cell lung cancer	Col4a1, Col4a2, Lamc3
map04010	MAPK signaling pathway	Map3k13, Cacna1g, Cacna1i, Flna

Appendix Table 5： Oxidative phosphorylation-related genes.

Pathway id	Description	Molecules
map00190	Oxidative phosphorylation	**Atp5e, Atp5h, Atp5j, Atp6v1g2, Cox5b Cox6c, Cox7a2, Gm10925, Gm12338 Gm28661, mt-Co1, mt-Cytb, mt-Nd1 mt-Nd2, mt-Nd4, mt-Nd5, mt-Nd6 Ndufa1, Ndufa4, Ndufa5, Ndufa6 Ndufb3, Ndufs4, Ndufs6, Ndufv3,Uqcrb**

Transplantation of MDD patient microbiota induces depressionlike behaviors in GF recipient mice

To investigate whether changes in gut microbiome contribute to the pathogenesis of MDD, FMT experiments were performed. This approach has been successfully used to determine the causative role of gut microbiota in the onset of obesity, colitis and type I diabetes. Here, as previously reported, adult GF mice were colonized with pooled fecal samples randomly derived from five non-medicated MDD patients and five healthy controls without a priori knowledge of their gut microbial profiles. In the post hoc 16S rRNA gene sequence analysis of these samples, PCoA plot of unweighted and

weighted UniFrac matrix showed that the gut microbial phenotypes of these randomly selected MDD and healthy control samples were similar to their corresponding groups. This humanized FMT model allowed us to: (i) determine whether the depressive phenotypes of MDD patients were transmissible via their gut microbiomes and (ii) comparatively analyze the gut microbial community structure, metabolism and host-microbial co-metabolism of the ‘depression microbiota’ and ‘healthy microbiota’ recipient mice. Initially, the weight of experimental mice was measured weekly.

We found that the weight of ‘depression microbiota’ recipient mice was not significantly different than 'healthy control' recipient mice during FMT experimentation. To test whether colonization of GF mice with ‘depression microbiota’ results in depression-like behaviors, three well-established behavioral tests—OFT, FST and TST—were performed on weeks 1 and 2 post FMT. On week 1 post FMT, there were no significant differences in OFT, FST and TST between ‘depression microbiota’ and ‘healthy microbiota’ recipient mice. However, on week 2 post FMT, the ‘depression microbiota’ recipient mice displayed a decreased proportion of center motion distance in the OFT and an increased duration of immobility in the FST and TST as compared with those of 'healthy control' recipient mice. However, there were no significant differences in total motion distance between the two groups in the OFT. These findings show that colonization of GF mice with ‘depression microbiota’ resulted in increased depression-like behaviors as compared with colonization with ‘healthy microbiota’. Given that anxiety is a common symptom among MDD patients, anxiety-like behavior was also tested here. We found that ‘depression microbiota’ recipients displayed a decreased center motion distance in the OFT, which is indicative of anxiety-like behavior. Therefore, in this humanized depressed model, diagnostic depressive behavior (as measured via the FST and TST) as well as non-diagnostic anxiety behavior (as measured via the OFT) associated with depression were transmissible via the gut microbiome.(Zheng P et al., 2016)

References:

Zheng P, Zeng B, Zhou C, Liu M, Fang Z, Xu X, Zeng L, Chen J, et al. (2016), Gut microbiome remodeling

induces depressive-like behaviors through a pathway mediated by the host's metabolism. Mol Psychiatry 21:786-796.
